# Nurses’ Self-Assessment of Caring Behaviors in Nurse–Patient Interactions: A Cross-Sectional Study

**DOI:** 10.3390/ijerph17145255

**Published:** 2020-07-21

**Authors:** Jasenka Vujanić, Nada Prlić, Robert Lovrić

**Affiliations:** 1 Faculty of Dental Medicine and Health Osijek, Josip Juraj Strossmayer University of Osijek, 31 000 Osijek, Croatia; nprlic@mefos.hr (N.P.); rlovric@fdmz.hr (R.L.); 2 Faculty of Medicine, Josip Juraj Strossmayer University of Osijek, 31 000 Osijek, Croatia

**Keywords:** caring, caring behaviors, clinical practice, nurses, nurse-patient relationship, humanism

## Abstract

Nurse–patient interactions based on caring behaviors ensure better working conditions and better-quality healthcare. The aim of this quantitative study is to examine how nurses self-assess the frequency of applying caring behaviors in nurse–patient interactions and to identify the differences in the application frequency of caring behaviors in relation to work experience and education level. The respondents were Bachelor of Science (BSc) nurses and nurses with basic training (VET) employed in different clinical departments of the Clinical Hospital Center in Croatia. The survey used the “Caring Nurse–Patient Interactions Scale (Nurse Version)”. The respondents assessed the caring behaviors from the subscale “needs” as the most frequently applied (median (Me): 4.7; interquartile range (IQR): 4.4–4.9), while the least frequently applied were the procedures from the subscale “sensitivity” (Me: 3.8; IQR: 3.2–4.3). The VET nurses reported applying caring behaviors to the subscales “hope” (*p* < 0.001), “problem-solving” (*p* = 0.003), and “environment” (*p* = 0.021) more frequently than BSc nurses did. Compared with less experienced respondents, the respondents with more than 30 years of work experience applied the caring behaviors on the subscales “sensitivity” (*p* = 0.009), “expression of emotions” (*p* = 0.001), “problem-solving” (*p* = 0.008), and especially “humanism” and “spirituality” (*p* < 0.001) more frequently. The results indicate that respondents are more focused on applying skills or carrying out a task than on caring behaviors which is about demonstrating compassion, loving kindness, and relationships.

## 1. Introduction

Nursing, like other healthcare professions, is based on the ideals of service to humanity [1,2]. Nurse–patient interaction implies a professional and therapeutic relationship based on the planning, provision, and assessment of caring that satisfies a patient’s individual needs [2]. Caring is an important part and aspect of providing nursing services and each nurse has the responsibility to develop and improve nursing services through caring behavior [3].

The importance of nurse–patient interactions based on caring behaviors has been proven in philosophical discussions, theories, and innovative research by the renowned theoreticians Watson, Leininger, Boykin, and Swanson [4,5,6]. These theoreticians consider caring to be the essence of nursing and the key element of both effective nurse–patient interactions and high-quality healthcare. Furthermore, health sciences are incomplete without caring science [4,5,6,7,8]. Even though Leininger and Watson have their own original nursing theories and ideas about nursing concepts for the year 2050, they both view caring as the essence of nursing [9].

From an objective perspective, caring as a concept is hard to define. Caring is what patients expect and should experience to be satisfied with their provided nursing services [3,10,11,12]. Many authors believe caring to be the essence which is to gain additional knowledge in clinical and research practices [13,14] and a key concept of evidence-based nursing practices [15]. Implementation of specific nurse–patient interaction models based on caring behaviors into healthcare systems could improve working environments, provide a higher level of satisfaction in both nurses and patients [12,16], assure a higher level of patient safety, and ensure high-quality healthcare and better economic conditions [2,3,11,13]. However, some authors warn that today’s caring behaviors are low [3,12,13,17,18]. Technological advancements have significantly altered the role of nurses and their interactions with patients and other healthcare professionals [12,19]. The impact of modern technology on healthcare varies a lot. Some authors believe that highly sophisticated technology makes the healthcare more impersonal and less humane, while others describe its neutral or even positive effects on patient satisfaction [20]. The undeniable fact is that the usage of modern medical gadgets (e.g., computer-based patient monitors, computer files, telehealth etc.) makes the healthcare process easier and reduces the risk of human mistakes which happen mostly due to the lack of nursing staff and the increased scope of their work [20,21]. For those reasons, technological advancements contribute to patient safety and reduce expenditure in healthcare systems on the one hand, but on the other hand they can reduce face-to-face interactions and impair the human element of healthcare [22]. In the study by Coatsworth-Puspoky et al. [23], patients state that hospital technologies and procedures separate them completely from the world they live in, and they wish nurses would communicate more with them. Therefore, while aiming at providing better quality healthcare and reaching higher levels of patient satisfaction, nurses have to find and maintain the proper balance between applying modern technology and a human approach to patients.

Carrying out tasks and reducing expenditure have become a priority in contemporary healthcare. Although nurses may feel as if they are being caring, they often provide healthcare while not actually taking care of what patients expect and want [24]. In addition, relevant studies show that nurses often neglect caring behaviors in interactions with their patients. Instead, they prefer the application of psychomotor competences in order to perform the task technically more efficiently [25,26]. Nurses’ low-level of affective care and lack of individual and humane approach are partially a consequence of their heavy workloads caused by additional work (e.g., administration) [12,24,25,26,27,28,29]. If more importance is given to physical aspects of care than to social or spiritual aspects, a patient becomes an object, which is not in compliance with the philosophy, values, legacy, theories, and professional attitudes of nursing [1]. Watson warns that such a trend could result in patient dissatisfaction with nursing care, consequently posing a real threat to the quality of the whole healthcare system [1]. Felgen at al. [30] emphasize that the recipients of nursing care expect to be treated humanely and become satisfied and loyal clients if they experience humane treatment through nursing care.

Moreover, the relevant literature suggests that the perception and implementation of caring behaviors are influenced by factors such as knowledge, formal education [31], and length of direct work with patients [3,32,33]. The importance of the early exposure to the content of caring behavior in formal nursing education is emphasized since future nurses develop their caring attributes at that early phase [34], as well as later during their work [19,35]. Nursing students learn and acquire competencies for implementing holistic healthcare through clinical education and personal experiences of learning in educational settings supported by competent teaching staff and their developed culture of providing healthcare. Although the clinical environment has a significant role in developing caring competences, it can also be an obstructive factor when clinical mentors, managers or other medical workers do not care about the culture of providing healthcare. Recent literature describes how education and work experience correlate with the level of caring attitudes and behaviors. For example, in 2019 Compton, et al. [36] described a positive correlation between education and the application of caring attitudes and behaviors, while other authors do not find any clear correlation [14]. It is evident that professional environments whose management is based on business economics and strictly task-oriented provide very little in terms of the humane approach to caring [37].

Generally, there is a difference in nursing between the human caring models, which are primarily aimed at holistic patient care and the reason why many nurses choose this profession, and the biomedical model, which is focused on task completion and maintaining the reality of institutional demands [14,38]. Nurses in the biomedical model often have a subordinate role and carry out doctors’ orders [28], which limits their autonomy in making decisions and in carrying out procedures, consequently leading to care and caring behavior becoming secondary. Development of nursing as a scientific discipline is based on a clear distinction between nursing and medical cognition, in which a nurse theorist, Jane Watson, had a huge role. In her Theory of Caring, Watson insisted on separating care from treatment and returning the nursing profession to its essential roots and values [25], which resulted in a new meaning of nursing as a separate theoretical paradigm in later phases of nursing development. Although the biomedical model is still dominant, caring models are becoming increasingly accepted [14].

The theoretical framework for this study is based on Watson’s human caring theory [1]. In Watson’s Theory of Caring, nursing is “concerned with promoting health, preventing illness, caring for the sick and restoring health”. Watson believes that holistic healthcare is central to the practice of nursing and defines nursing as “a human science of persons and human health-illness experiences that are mediated by professional, personal, scientific, esthetic, and ethical human transactions.” It places the patient in the context of the family, community, and culture, and the focus of the practice is on the patient rather than the technology. Human caring describes the attitudes and behaviors that demonstrate interest in and respect for patients’ psychological, social and spiritual concerns and values. Humane caring develops as a specific competence in stages through nursing education and clinical practice, in parallel with other nursing competencies such as ‘act in a professional manner’, ‘clinical reasoning in nursing’ and ‘clinical nursing leadership’ [8].

The essence of Watson’s theoretical contribution is captured in 10 carative factors. The first three, in her own words, are the philosophical foundation for the science of care [8]. Table 1 presents the 10 carative factors and their descriptions.

Each factor contains a dynamic phenomenological component related to each individual in nursing care. Through these carative factors, nurses perform their basic professional duty. Watson’s carative factors are well-accepted in the profession because they express the humanistic value of care. Watson promoted the concept of 10 carative factors during the clinical caritas processes [1,7]. These processes redefined the factors in action-oriented ways to guide caring practices. The carative factors/caritas processes serve as structure and order for a theoretical philosophical foundation for the discipline and profession of nursing. Finally, the exceptional contribution of Watson’s theory lies in the fact that it combines many approaches of conceptualized caring into one source, as well as in developed instruments that assess the quality of caring and nurse–patient interaction. Watson’s focus on caring and humanism has motivated researchers to study the caring concept in more depth.

Caring cannot be measured or quantified objectively [13]. Still, specific measuring instruments can be useful as supplementary tools for assessment and self-assessment of high-quality nurse–patient interaction from the perspective of caring behaviors [13,29,39,40].

In 2005, Cossete developed a Nurse–Patient Interaction Scale (CNPI-70) as a reliable instrument to assess the quality of nurse–patient interactions (quality of care). The CNPI-70 consists of 70 items and 10 carative factors/subscales based on Watson’s Theory of Caring [41]. All 70 items describe a respondent’s attitudes towards behaviors regarding nursing care in clinical practice that can be measured in terms of importance, frequency, and applicability (Table 2). The formulation of the items varies according to the target group, i.e., the respondents. Due to its items and their relevance and comprehensiveness, CNPI-70 is applicable for various groups of respondents (e.g., patients, family members, nurses, nursing students) [25,33,41].

A Philippine study by Fortuno [12] used the CNPI-70 scale to assess the prevalence of caring behaviors by nurses in nurse–patient interactions. The results showed that nurses most commonly apply care procedures from two carative factors: “humanism” and “environment”. Furthermore, the CNPI-70 scale was used in a Swiss study to assess caring behaviors as perceived by hemodialysis patients and their nurses [42]. For patients the carative factor “needs” was more important than for nurses who consider the factor “environment” to be more important [42]. Patients and nurses assessed the carative factor “problem-solving” to be the least important among all 9 carative factors. Another comparative descriptive study carried out in Switzerland in 2019 used the CNPI-70 scale to examine and compare the quality of caring attitudes and behaviors as perceived by hemodialysis patients and their nurses [43]. The results showed that both nurses and patients reported a high prevalence of caring attitudes and behaviors, while patients assessed all carative factors, except “spirituality”, higher than nurses [43]. In the same year, another descriptive study was carried out in Switzerland. Its aim was to describe and compare nurse and inpatient perceptions of caring attitudes and behaviors in rehabilitation [15]. The results showed that perceptions of caring were assessed highly, especially by nurses. Patients assessed highly items related to clinical aspects of caring, while nurses considered humanistic and clinical aspect of caring as equally important. In both groups, comfort care was important, while relational caring items were least important. The CNPI-70 scale was used in an American study whose aim was to examine the impact of several carative factors demonstrated by home health nurses [32]. The results showed that the care theory principles were widely practiced in home health settings, while regression analysis showed that age, years of experience, and education/credentials increased, the total caring score on the CNPI-70 survey similarly increased.

The relevant literature identifies nurses’ level of education and exposure to care as key personal factors that affect perception and application of caring behaviors [3,31,32,33]. Thus, these factors are used as criteria in this study.

It can be noticed that there is still an insufficient number of studies that have researched the nursing perception of caring behaviors in nurse–patient interaction using the CNPI-70 scale. This deficit affects the global understanding of the importance of caring behaviors and the importance of the ability to self-assess in order to make personal and professional progress, as well as consequently to improve whole organizations [28]. Therefore, the aim of this study was to overcome this literature gap by providing results specifying the nursing perception of applying caring behavior in their clinical practice.

The aim of this study was to research how nurses self-assess the frequency of applying caring behaviors in nurse–patient interactions and to identify the differences in application frequency of caring behaviors in relation to work experience and education level.

## 2. Materials and Methods

### 2.1. Study Design

This cross-sectional study was conducted in the Clinical Hospital Center in Croatia (CHC). The quantitative research approach was used and an anonymous survey was performed using a closed-ended questionnaire.

### 2.2. Respondents

Respondents were selected using the principle of availability according to the defined criteria. Thus, the study included a total of 735 registered nurses who are permanently employed at the clinics and departments, and who are in direct contact with patients and provide 24 h healthcare. According to Watson [1], the constant presence of a nurse is a precondition for developing and maintaining trust, effective caring behavior, consciousness, dedication, and authenticity in nurse–patient relationships. This is why this study included nurses with high school vocational education and training (VET) and Bachelor of Science (BSc) nurses who provide continuous and direct healthcare. Nurses who had brief interactions with patients (e.g., nurses in the outpatient ward, operating block, emergency reception, department of sterilization, transfusiology, intrahospital infections, quality departments) were not included in this research.

The sample size was calculated using the online software Sample Size Calculator from Creative Research Systems [44]. The calculation was based on the total number of CHC nurses (1192) with an initially defined confidence interval value of 3%, confidence level of 95%, and α level of 0.05 [44]. According to this study’s calculations, the lowest sample size required was 563 respondents.

### 2.3. Instrument

Data were collected using the translated and standardized 70-item version of the Caring Nurse–Patient Interactions Scale Questionnaire (Nurse Version; CNPI-70) [41]. This version was designed by Cossette, Cara, Ricard, and Pepin in 2005 on the basis of Watson’s caring theory [41]. The questionnaire items represent carative factors and are divided into 10 subscales: “humanism”, “hope”, “sensitivity”, “helping relationship”, “expression of emotions”, “problem-solving”, “teaching”, “environment”, “needs”, and “spirituality” [41]. The authors are aware of Watson’s recent work on clinical caritas processes and the danger of reducing the wholeness of caring. However, both the French and English versions of the CNPI have been useful not only in a research context but also for clinical and educational purposes [25]. The results of a psychometric study conducted using this questionnaire showed a strong potential for use in research in clinical and educational facilities [41]. Cronbach alpha values for each of the 10 subscales ranged from 0.73 to 0.91 [40].

The CNPI-70 questionnaire was translated from English to Croatian through the following steps: forward translation by two bilingual experts independently; back translation, without any reference to the original instrument wording; comparison of the original and the translated items by another bilingual expert; and review of the translated questionnaire to comply with the system standards for clinical practice. The reliability of both questionnaires was tested using Cronbach’s coefficient. The reliability of each CNPI-70 subscale ranged from 0.75 (“humanism”) to 0.90 (“problem-solving”). The overall CNPI-70 questionnaire reliability was 0.97, indicating a high reliability. To examine the frequency of caregiving in clinical practice, the question was highlighted at the beginning of the questionnaire: “How frequently do you apply the attitudes and behaviors described in each of the following statements?” The self-rating scale used a five-point Likert scoring system. Each item was scored from 1 to 5 points (almost never = 1, sometimes = 2, often = 3, very often = 4, almost always = 5).

### 2.4. Data Collection

Data were collected for four months at clinics/departments from the aforementioned institution. The researchers (authors of this manuscript) distributed a questionnaire in the clinical departments to all nurses. Nurses were invited to participate voluntarily in this study by completing and returning the questionnaire in sealed envelopes. Respondents completed the questionnaires using the pencil-paper method. The time for completing the questionnaire was not limited; it lasted on average 30 min. The respondents voluntarily and willingly engaged in the research, as they have witnessed great changes in nursing in the Republic of Croatia. Before each data collection, the researcher thoroughly explained the research’s purpose, the ethical issues, and the questionnaire’s details to the respondents. The respondents had the right to withdraw before and during the questionnaire completion. The anonymity of the participants was guaranteed, and there was no possibility to determine their identity from the responses. Only researchers had access to research data.

### 2.5. Data Analysis

Statistical analysis was conducted using SPSS Statistics for Windows, (Version 17.0. for Windows, SPSS Inc., Chicago, Illinois, USA). *p* < 0.05 was considered statistically significant. Descriptive statistics for nominal variables were expressed as proportions and percentages. The normality of the distribution of numerical variables was tested by the Shapiro–Wilk test. Numerical variables (age, work of experience, self-assessments of respondents) are not following a normal distribution and are represented by the median (Me) and interquartile ranges (IQR). Therefore, the non-parametric Kruskal–Wallis test was used to compare the median differences among several groups, while the Mann–Whitney U test was used to compare the median differences between the two groups. The statistical analysis of the reliability of each scale/subscale and the overall CNPI-70 questionnaire was conducted using Cronbach’s alpha coefficient.

### 2.6. Ethical Consideration

The author gave us consent for the translation and use of the CNPI-70 instrument. This study was approved by the Institutional Review Board of the CHC (IRB approval number: R1:8099-7).

## 3. Results

Of the 735 distributed questionnaires, 697 (94.8%) were valid, thus satisfying the calculated minimal number of respondents. Thus, the study included 697 respondents, of whom 642 (92.1%) were female and 55 (7.9%) were male. The age of the respondents ranged from 19 to 65, with a median (Me) of 37 years (IQR 30–48). Regarding education level, 533 (76.5%) respondents completed VET and 164 (23.5%) completed their bachelor’s degree. Years of service in nursing ranged from 1 to 44, with a median (Me) of 17 years (IQR 9–27).

**Figure 1:**
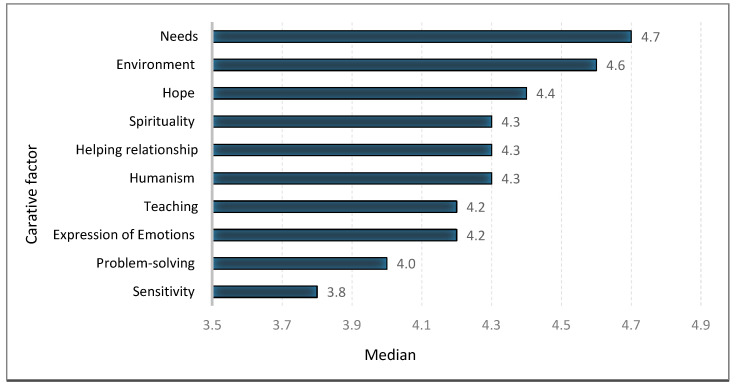
Nurses’ self-assessment of the frequency of application of caring behaviors.

Of the 10 questionnaire subscales, the highest median score was 4.7 (IQR 4.4–4.9) and this was achieved for the subscale “needs”, while the “sensitivity” subscale received the lowest median score (3.8) (IQR 3.2–4.3) (Figure 1 and Table 3).

Respondents with up to 15 years of work experience (*n* = 307) estimated that they apply significantly less the caring behaviors from the following subscales: “humanism” (*p* < 0.001), “sensitivity” (*p* = 0.009), “expression of emotions” (*p* = 0.001), “problem-solving” (*p* = 0.008), and “spirituality” (*p* < 0.001) compared to respondents with more than 30 years of work experience (Table 3).

The VET nurses reported applying caring behaviors related to the subscales “hope” (*p* < 0.001), “problem-solving” (*p* = 0.003), and “environment” (*p* = 0.021) more frequently than BSc nurses did (Table 4).

## 4. Discussion

The aim of this study was to examine how nurses self-assess the frequency of application of caring behaviors in nurse–patient interactions, especially considering education level and work experience.

The results showed that respondents self-assessed certain subscales in a range from 3.8 to 4.7. Considering the range and score interpretation on the Likert’s scale (from 1 to 5) of the CNPI-70 instrument, respondents self-assessed the application of certain caring behaviors as very often or almost always. High subscale scores were described in the study by Delmas et al. [43] despite their scores for certain subscales being significantly lower (2.88 to 4.31). Other studies describe completely opposite results with very low scores of caring behaviors indicating dehumanization of care [45,46].

Despite the high self-assessment score in this study, data analysis showed a significant difference in the frequency of application in certain caring behaviors. Therefore, respondents gave the highest self-ratings for the subscales “needs” (4.7) and “environment” (4.6), which was only partially supported by the results of other relevant studies [12,42,43]. A Swiss study showed that nurses gave the highest score on the “environment” subscale (4.4), while patients gave the highest score on the “needs” subscale (4.3) [42]. Both subscales were scored relatively highly by nurses (4.09–4.11) in a later Swiss study, although the subscale “humanism” (4.31) received the highest score [43]. Moreover, the “environment” subscale was scored high (4.47) by nurses in the Philippine study, while the “humanism” subscale (4.56) scored the highest [12]. As a result of this, this study respondents defined their patient interaction mostly on providing healthcare to satisfy human needs [25]. These results were expected since nursing education in Croatia is hugely based on Henderson’s theory of basic human needs [47]. Henderson and Watson believe that nursing should take a holistic approach, stating that nursing is not only supposed to fulfill a patient’s physical needs but also satisfy a patient’s psychosocial, social, and spiritual needs. While Henderson focuses primarily on providing complete help to persons to satisfy their basic human needs, Watson’s theory is based on caring relationships with individuals, especially on the spiritual aspects of a person’s life [25]. Furthermore, a high score for the “environment” subscale indicated that respondents attached importance to internal and external environmental factors that affect health and consequently the disease of an individual. The concepts relevant to an internal environment include mental and spiritual wellbeing, as well as social and cultural beliefs. Aside from epidemiological variables, other external factors include comfort, privacy, safety, and a clean/aesthetic environment [25].

It is evident that respondents respect the concept of basic human needs. However, the highly assessed subscales (“needs” and “environment”) belong to the clinical aspects of care [40]. By contrast, subscales “hope”, “humanism”, “helping relationship”, “spirituality”, and “expression of emotions” scored the lowest (4.2 to 4.3), which is supported by the results of a Swiss study in which nurses rated the environment subscale before humanism [43]. However, the results of this study differ from the aforementioned relevant studies in which nurses, using the CNPI-70 scale, ranked the “humanism” subscale the highest [12,42,43,48]. This subscale favors the humanistic–altruistic system of values, wherein one experiences satisfaction and fulfills personality by giving themselves to others [34]. Although the aforementioned values are acquired at an early age, they are significantly shaped later under the influence of various formal and informal factors (e.g., influence of a mentor, educator, etc.) Moreover, the “hope” subscale, which Watson placed together with “humanism” and “sensitivity” in a philosophical foundation for the science of care, was also ranked lower in this study than subscales from the clinical aspects of care, which was supported by the results of other studies [12,42,43]. According to Watson [1], the “hope” subscale promotes holistic nursing care, defines nursing roles while developing a desired nurse–patient relationship, and promotes a patient’s acceptance of positive health behaviors [41]. Thus, these results show a clear direction towards clinical skills among nurses, which can be explained by the daily increase in the scope of nurses’ service, patients’ shorter stays on wards, and the insufficient number of nurses. Similar problems have been mentioned in other studies [24,26,27,28,41]. Hence, nurses in clinical practice “must do the job” and be effective, and thus they often do not have the time to provide effective care [24,26,27,28,41]. Many authors describe difficult working environments and inefficient organizations as the common external factors that negatively impact acquisition and application of caring behavior, thereby stimulating the lack of individual and humane approaches [24,26,27,29].

According to Fortuno [12], key factors that negatively affect the acquisition and application of caring behavior include the fast pace in hospitals, nursing services aimed at goals and tasks, and working overtime. Aggravating circumstances, such as the obligation to document all procedures to prevent possible lawsuits, and extensive laboratory examination and treatments, shorten communication time with patients and the provision of necessary healthcare [12]. Moreover, the authors of a Chinese study identified the problems of staff shortages and an onerous workload to be the reasons why nurses believe that expressive caring activities are less feasible compared with operational caring behavior [26]. According to Watson [1], caring behavior can be expressed through emotional and operational/technical activities. Emotional activities are those that affect patient mood, such as honesty, trust, hope, and compassion. Operational activities include satisfying basic human needs and assuring patient comfort through activities such as reducing pain, administering medications, ensuring patient safety, etc.

In this study, the “problem-solving” and “sensitivity” subscales were rated lowest, which is in full accordance with the results of other studies [12,42,43]. For example, Delmas [43] found that nurses rated extremely low two subscales: “problem-solving” (2.66) and “sensitivity” (2.33). Furthermore, the Philippines study also stated that nurses ranked these two subscales the lowest according to their importance and frequency of use [12]. Unfortunately, such results suggest that nurses are less focused on their relationship with patients and more focused on other aspects of caring. If nurses do not accept their own sensitivity and feelings, they can easily become less honest, open, and sensitive towards others [12]. However, constant exposure to trauma may alleviate feelings of empathy for the patients they provide for. Moreover, administrative duties, communication with a patient’s family, and tending to a patient’s psychological needs put additional strain on nurses, resulting in further loss of compassion, social exhaustion, and ineffective communication, which is mostly manifested in a lack of answers to patients’ questions when patients need answers [3,24]. Many authors suggest that the situation could improve through providing better staff education and peer support, effectively allocating staff, and promoting a democratic work environment [3,32,33,49].

In this study, the respondents with up to 15 years of work experience rated procedures related to “humanism”, “sensitivity”, “expression of emotions”, and “problem-solving” significantly lower compared to the respondents with over 30 years of work experience. These findings are supported by the results of other studies [25,49]. Vandenhouten et al. [50] analyzed caring behaviors according to age, gender, and years of practice, and the results showed a significant difference between the perception of caring in older female nurses (age 50 and older) and in nurses with 20 or more years of work experience, who had higher caring scores [51]. The results of a study conducted in Shanghai showed that age, years of work experience, and position greatly affected caring behavior, especially regarding competency and feasibility [26]. The authors explained that age, work seniority, and promotion to teacher or head nurse positions implied a rich life experience, accumulation of work experience, and improvements in various abilities. These conclusions are in accordance with Watson’s theory (the latest book), which states that caring ability is related to values, professional knowledge structure, and work experience of nurses [26]. In addition, the results of the recent study by Lechleitner [33] indicate that as age and experience increased, so did the total caring score. The authors explained the results with the fact that people can become more considerate towards others as they grow older or gain new experiences. Their increased care could also indicate their need for high-quality care as they grow older [33].

The results of our study show that respondents with more years of work experience valued spirituality more highly than the respondents with fewer years of work experience. According to Watson [1], spiritual care is as important as physical needs. Nurses must identify caring moments with their patients. According to research by Murray and Dunn [52], most nurses stated that their education and training had not prepared them to provide adequate spiritual care for their patients. There was a statistically significant increase in knowledge, self-confidence, and competence in nurses who had attended a workshop on spiritual care [52]. Other authors imply that there is insufficient knowledge and training regarding nurses’ perceptions and nursing practice related to spiritual care, which results in general confusion [53,54].

With regard to education, BSc nurses in this study assessed the “hope”, “problem-solving”, and “environment” subscales lower than nurses with basic training (VET). These results can be explained using specific organizational models in clinical practice in which BSc nurses are oriented toward organizational work, management, administrative work, and communication with other healthcare professionals. Thus, these nurses have less time for direct interaction with their patients. There are no significant differences regarding education in relation to other carative factors. Some studies point out the positive correlation between education level and caring attitudes and behaviors [36], while other studies do not describe any clear correlation between carative factors and a nurse’s education level [14]. However, some authors state that there is a tendency to reduce caring behavior without any connection to nurses’ education level [55,56]. The situation in which levels of caring behavior are inversely proportional to the years of working experience is a phenomenon that can be the consequence of many negative factors in everyday clinical practice (e.g., experience damaged relationships, negative supervisory relations, poor cooperation and deficient communication among team members) [37].

According to Watson [1], the problem-solving factor is related to the systematic use of scientific methods of creative problem-solving as the basis for decision-making or the application of healthcare processes and the scientific approach to problem-solving [7]. There are no differences in relation to the education or teaching carative factors, nor in relation to the promotion of mutual teaching and learning. This is an essential factor in nursing because it constitutes a difference between nursing care and medical treatment [7] Jiang et al. [26] noted that nurses with higher education have higher competence levels and feasibility results. Vandenhouten et al. [50] showed that there were no significant differences in providing caring behavior with respect to a nurse’s education level. These authors assume that nurses familiar with Watson’s theory of transpersonal care would better observe their own application of caring behaviors [50]. It is necessary to stress the significance of caring theories during initial nursing education as well as during their further education. Nurses need lifelong education and training in order to uphold the essential nursing values and ensure that caring remains vital in their nursing practice [51].

### 4.1. Further Research and Practice

We believe that it is important to focus subsequent research on nursing students and their patients to determine which caring behaviors are considered more significant from the perspective of patients, students, and registered nurses. Furthermore, additional longitudinal studies using various research methods (i.e., quantitative, qualitative, and mixed) should be carried out in order to identify the impact of carative factors on patient satisfaction. Also, future studies should research the correlation between nursing caring behaviors and the patient outcomes. Moreover, it would be useful to research the effect of time on developing and maintaining the relationship of trust with a patient for the purpose of effective caring behavior.

### 4.2. Limitations of the Study

The study had certain limitations. First, research was conducted in a single clinical hospital, and we believe that it should be extended to other clinical centers to gain an overview of application of caring attitudes and behaviors. Second, this study’s results which directly relate to specific items in CNPI-70 scale were compared to only a few available studies which had used CNPI-70 scale for assessing importance and frequency of application of nursing caring attitudes and behavior in nurse–patient interactions. Third, since self-assessment was carried out using the CNPI-70 scale, respondents may have inflated their scores to be viewed as a more caring health professional [40].

### 4.3. Implications for Nursing

This study contributes to the dissemination of knowledge related to caring behavior and a better understanding of nurse–patient interactions on a global level. The results showed that caring behaviors are vital segments in nurse–patient interactions, and their quality needs to be assessed and self-assessed. The CNPI-70 scale can be of immense help to nurses in clinical practice to identify the areas of strength and weakness in nurse–patient interactions, which will lead to self-correction and improve the relationship with patients. Furthermore, a better quality of nurse–patient relationship can improve working conditions, improve patient safety, and ensure a higher level of satisfaction for both nurses and patients, thus providing significantly better healthcare. Moreover, the results of this study can be used to analyze and review the existing nursing curricula and to design curricula that will include the content of caring behaviors so that nursing students will learn their importance through efficient methods.

## 5. Conclusions

Based on the study results, respondents most frequently apply caring behaviors related to assistance with gratification of human needs (“needs”), whereas cultivation of sensitivity to one’s self and others (“sensitivity”) is the most rarely applied. Moreover, there are differences in the application of attitudes and caring behaviors in clinical practice according to years of experience and education level. BSc nurses in this study assessed the subscales “hope”, “problem-solving”, and “environment” lower than nurses with basic training (VET). Respondents with up to 15 years of work experience assessed factors related to “humanism”, “sensitivity”, “expression of emotions”, and “problem-solving” significantly lower than those with more than 30 years of work experience. The results indicate that respondents are more focused on applying skills or carrying out a task than on caring, which is about demonstrating compassion and lovingkindness in their professional relationships.

## Figures and Tables

**Table 1:** Carative factors, their descriptions.

Carative Factor (CF)	Description
1. Humanism	Formation of a humanistic-altruistic system of values. Humanistic-altruistic feelings and acts provide the basis of human caring and promote the best professional care, and as such, constitute the first and most basic factor for science and ethic of caring.
2. Hope	Instillation of faith-hope. In this carative factor (CF), patient’s beliefs are encouraged, honored and respected as significant influences in promoting and maintaining health.
3. Sensibility	Cultivation of sensitivity to one’s self and to others. Nurses who recognize and use their sensitivity promote self-development and self-actualization, and are able to encourage the same growth in others. Without this factor nursing care would fall.
4. Helping relationship	Development of a helping-trusting, human caring relationship. The human caring relationship is transpersonal. In that it connotes a special kind of relationship: a connection with the other person, a high regard for the whole person and their being in the world.
5. Expression of emotions	Promotion and acceptance of the expression of positive and negative feelings. The caring relationship can move to a deeper, more honest and authentic level if he nurse allows for this CF.
6. Problem-solving	Systematic use of a creative problem-solving caring process. This process involves full use of self and all of one’s faculties, knowledge, instincts, intuition, aesthetics, technology, skills, empirics, ethics, personal and even spiritual knowing.
7. Teaching	Promotion of transpersonal teaching-learning. This CF makes explicit that learning is more than just receiving information and data. It involves a caring relationship as context for any teaching learning.
8. Environment	Provision for a supportive, protective and/or corrective mental, physical, societal, and spiritual environment. The areas that involve this factor are: comfort; privacy; safety; clean; aesthetic surroundings.
9. Needs	Assistance with the gratification of human needs. All needs are equally important and must be valued and responded to for caring-healing.
10. Spirituality	Allowance for existential-phenomenological-spiritual forces. This CF allows for spiritual filled meanings and unknowns to emerge open to infinite possibilities for miracles.

**Table 2:** First items from 10 carative factors of the Caring Nurse-Patient Interactions Scale—Nurse Version (CNPI-70).

Carative Factor (Number of Items)	First Items
1. Humanism (1–6)	Treat them as complete individuals, show that I was interested in more than their health problem.
2. Hope (7–13)	Show that I will be there for them if they need me.
3. Sensibility (14–19)	Ask them how they would like things to be done.
4. Helping relationship (20–26)	Listen to them attentively when they speak, as well as those closest to them.
5. Expression of emotions (27–32)	Encourage them to speak their thoughts and feelings freely.
6. Problem-solving (33–38)	Help them to set realistic goals that take their health condition into account.
7. Teaching (39–47)	Help them to identify, formulate and ask questions about their illness and its treatment.
8. Environment (48–54)	Understand when they need to be alone.
9. Needs (55–64)	Help them with the care they cannot administer themselves.
10. Spirituality (65–70)	Help them to feel well in their condition.

**Table 3:** Scope of carative factors in relation to the respondent’s years of experience.

Carative Factors (F1–F10)	Years of Experience Median (Interquartile Range)				*p* *
	≤15 years	16–30 years	>30 years	Total	
	*n* = 307	*n* = 287	*n* = 103	*n* = 697	
F1—Humanism	4.2 (3.5–4.7)	4.3 (3.8–4.7)	4.3 (4.0–4.8)	4.3 (3.7–4.7)	<0.001
F2—Hope	4.4 (3.9–4.9)	4.4 (4.1–4.9)	4.6 (4.0–4.9)	4.4 (4.0–4.9)	0.072
F3—Sensitivity	3.7 (3.2–4.3)	3.8 (3.3–4.3)	4.2 (3.5–4.3)	3.8 (3.2–4.3)	0.009
F4—Helping relationship	4.3 (3.6–4.7)	4.4 (3.8–4.7)	4.4 (3.9–4.7)	4.3 (3.7–4.7)	0.128
F5—Expression of emotions	4.0 (3.5–4.7)	4.3 (3.7–4.7)	4.3 (4.0–4.8)	4.2 (3.7–4.7)	0.001
F6—Problem solving	3.8 (3.3–4.5)	4.0 (3.5–4.7)	4.2 (3.7–4.7)	4.0 (3.5–4.7)	0.008
F7—Teaching	4.2 (3.6–4.7)	4.3 (3.8–4.7)	4.4 (4.0–4.7)	4.2 (3.7–4.7)	0.055
F8—Environment	4.6 (4.1–5.0)	4.6 (4.0–4.9)	4.7 (4.3–5.0)	4.6 (4.1–4.9)	0.491
F9—Needs	4.7 (4.3–4.9)	4.7 (4.4–4.9)	4.7 (4.5–4.9)	4.7 (4.4–4.9)	0.311
F10—Spirituality	4.2 (3.5–4.7)	4.5 (3.8–4.8)	4.4 (4.0–4.8)	4.3 (3.8–4.8)	<0.001

* Kruskal–Wallis test

**Table 4:** Scope of carative factors in relation to the education level.

Carative Factors (F1–F10)	Education Level Median (Interquartile Range)			*p* *
	Vocational-level	Bachelor’s Degree	Total	
	*n* = 533	*n* = 164	*n* = 697	
F1—Humanism	4.3 (3.7–4.7)	4.3 (3.7–4.9)	4.3 (3.7–4.7)	0.664
F2—Hope	4.5 (4.0–4.9)	4.4 (3.9–4.8)	4.4 (4.0–4.9)	<0.001
F3—Sensitivity	3.9 (3.3–4.4)	3.8 (3.0–4.3)	3.8 (3.2–4.3)	0.343
F4—Helping relationship	4.4 (3.7–4.7)	4.3 (3.6–4.7)	4.3 (3.7–4.7)	0.061
F5—Expression of emotions	4.2 (3.6–4.7)	4.2 (3.7–4.7)	4.2 (3.7–4.7)	0.080
F6—Problem solving	4.0 (3.7–4.7)	3.9 (3.3–4.6)	4.0 (3.5–4.7)	0.003
F7—Teaching	4.2 (3.7–4.8)	4.2 (3.8–4.9)	4.2 (3.7–4.7)	0.256
F8—Environment	4.6 (4.2–4.9)	4.5 (4.1–4.9)	4.6 (4.1–4.9)	0.021
F9—Needs	4.7 (4.5–4.9)	4.6 (4.2–4.9)	4.7 (4.4–4.9)	0.161
F10—Spirituality	4.3 (3.8–4.9)	4.3 (3.7–4.8)	4.3 (3.8–4.8)	0.114

* Mann-Whitney test.
